# Combined Strategy of Endothelial Cells Coating, Sertoli Cells Coculture and Infusion Improves Vascularization and Rejection Protection of Islet Graft

**DOI:** 10.1371/journal.pone.0056696

**Published:** 2013-02-20

**Authors:** Yang Li, Wujun Xue, Hongbao Liu, Ping Fan, Xiaohong Wang, Xiaoming Ding, Xiaohui Tian, Xinshun Feng, Xiaoming Pan, Jin Zheng, Puxun Tian, Chenguang Ding, Xiaohu Fan

**Affiliations:** 1 Hospital of Nephrology, First Affiliated Hospital, Medical College, Xi’an Jiaotong University, Xi’an, China; 2 Department of Nephrology, Xijing Hospital, Fourth Military Medical University, Xi’an, China; 3 Department of Rheumatism and Immunology, First Affiliated Hospital, Medical College, Xi’an Jiaotong University, Xi’an, China; 4 University of Alberta, Edmonton, Alberta, Canada; Instituto Butantan, Brazil

## Abstract

Improving islet graft revascularization and inhibiting rejection become crucial tasks for prolonging islet graft survival. Endothelial cells (ECs) are the basis of islet vascularization and Sertoli cells (SCs) have the talent to provide nutritional support and exert immunosuppressive effects. We construct a combined strategy of ECs coating in the presence of nutritious and immune factors supplied by SCs in a co-culture system to investigate the effect of vascularization and rejection inhibition for islet graft. In *vivo,* the combined strategy improved the survival and vascularization as well as inhibited lymphocytes and inflammatory cytokines. In *vitro,* we found the combinatorial strategy improved the function of islets and the effect of ECs-coating on islets. Combined strategy treated islets revealed higher levels of anti-apoptotic signal molecules (Bcl-2 and HSP-32), survival and function related molecules (PDX-1, Ki-67, ERK1/2 and Akt ) and demonstrated increased vascular endothelial growth factor receptor 2 (KDR) and angiogenesis signal molecules (FAk and PLC-γ). SCs effectively inhibited the activation of lymphocyte stimulated by islets and ECs. Predominantly immunosuppressive cytokines could be detected in culture supernatants of the SCs coculture group. These results suggest that ECs-coating and Sertoli cells co-culture or infusion synergistically enhance islet survival and function after transplantation.

## Introduction

Islet transplantation (ITx) has become a popular treatment strategy for type I diabetes mellitus [1], [2]. Islet death includes β-cell loss during the culture period and after transplantation so that it is still an obstacle to successful ITx. The primary reasons for cell death include apoptosis and reduced β-cell function as a result of hypoxic stress and nutrient shortage during the isolation process and cell culture period [3].

The vascular system of the islet during the process of isolation and purification is destructed. The diffusion of oxygen and nutrition is only located at peripheral part of islets; however, β-cells located at the center of the islet are always in an ischemic state caused by apoptosis and dysfunction before angiogenesis [4]. Therefore, in the early stage of transplantation, the survival rate and islet functions depend on the timing and degree of revascularization [5]. In addition, immune rejection is still an important problem to limit the extensive application of islet transplantation. During the rejection process, immune cells and secreted inflammatory factors can cause direct death of islet cells [2]. Moreover, immune attack can delay the reconstruction of islet microcirculation, thus affecting the survival and functions of islets [6]. The strategies for improving islet vascularization and protecting from rejection need to be further explored.

The endothelial cell (EC) loss is one of the important reasons for delaying the reconstruction of microcirculation in islets [7]. In our previous studies, we have attempted to improve islet survival during islet transplantation through co-culture and co-transplantation of ECs [8], [9]. However, the distribution of ECs in these experiments is still on the peripheral part or surface of islets. The formed microvascular system cannot extend to the internal islet cells so that it is difficult to resolve the hypoxia of islet β-cells. A novel technique that isolated islets coating with primary human aortic ECs has been developed, which can generate a biologically active surface on the islets to inhibit instant blood-mediated inflammatory reaction (IBMIR) [10]. Since ECs on the surface of islets participate multiple step processes of angiogenesis, the islets coating with ECs may improve revascularization of islets themselves for decreasing the apoptosis of β-cells inside islets under the environment of hypoxia stress. However, the survival and integrity of islets are highly correlated with coating effect. The survival rate and functions of islets reveal an obvious decrease during this period [11]. Therefore, it is very important to keep morphological integrity of islets for ensuring the enough coating of ECs on the surface of islets.

Although coating can increase the number of ECs on the surface of islets, the proliferation speed of ECs on the surface of islets also can directly affect islet re-vascularization process because the angiogenesis needs time and the cultivation of islets requires 7–14 days. In addition, ECs have strong antigencity and are easy to be activated by inflammatory factors, which is the reason of rejection response and inflammatory reaction after islet transplantation [12], [13]. Therefore, the strategies for effectively improving coating efficiency and proliferation rate of ECs and reducing rejection response are critical points.

Sertoli cells (SCs) have been regarded as nurse cells in seminiferous tubules [14]. They can secrete many types of active proteins including IGF-1, EGF and bFGF as the nutrition sources for the growth and development of germ cells [15]. Our study has demonstrated that SCs as feeder layer can enhance the viability and function of co-cultured islet by regulating the expression of apoptosis-related gene and molecules [16], [17]. In addition, previous studies have also reported that SCs can promote the proliferation of ECs and gene expression of angiogenesis as well as reduce EC immunogenicity to lymphocytes [18]. Moreover, SCs can produce a wide variety of proteins including immunoregulatory factors such as Fas-L and TGF-β1 that are potent immunosuppressive factors, which can suppress the secretion of inflammatory factors in cells and protect the mouse and islet allografts from immune rejection [19]–[21]. Our previous study has demonstrated that the intravenous infusion of SCs can induce systemic immune tolerance with simple procedures, and the infusion can be repeated to further prolong the survival of grafts [22].

Based on improved isolation and purification of adult islets, the aim of our study is to construct vascularized islet grafts by an EC-coating method coupled with co-culture of SCs, which will prolong the survival time of islets during in vitro culture and promote vascularization process of islets by nutritional effect of SCs. In addition, the intravenous infusion of SCs can inhibit rejection response during islet transplantation depending on immunosuppressive effect. We hypothesize that the application of this combinatorial strategy may attenuate both short-term and long-term loss of islet grafts during pancreatic islet transplantation.

## Materials and Methods

### Ethics Statement

Sprague-Dawley rats and Wistar rats were provided by the Experimental Animal Center of Medical College of Xi’an Jiaotong University (Xi’an, China). This study was carried out in strict accordance with the Guidelines on the Care and Use of Laboratory Animals issued by the Chinese Council on Animal Research and the Guidelines of Animal Care. All procedures involving animals were approved by the Institutional Animal Care and Use Committees of the Xi’an Jiaotong University. All efforts were made to minimize animals’ suffering and to reduce the number of animals used.

### Isolation of Islets, Endothelial Cells and Sertoli Cells

Pancreatic islets, ECs and SCs were all isolated from Sprague-Dawley rats as described in our previous study [8], [22]. Pancreatic islets were isolated using collagenase P (Roche Diagnostics, Germany) digestion and discontinuous Ficoll (Type 400DL; Sigma, St. Louis, MO) gradient purification. Aortic ECs were isolated by incubation with collagenase II (1 mg/mL; Sigma, St. Louis, MO) followed by centrifugation at 1000 rpm for 10 minutes. After washing with PBS, the aortic ECs were resuspended in RPMI 1640 culture medium containing 10% fetal bovine serum (FBS, Gibco, Grand Island, NY). Briefly, SCs were separated from seminiferous tubules to a two-step sequential enzymatic treatment at 37°C with trypsin (Sigma Chemical Co.) and DNAase (Sigma Chemical Co.) in the first step and with collagenase P and hyaluronidase (Sigma Chemical Co.) in the second step. Then SCs were isolated by and then subjected to hypotonic treatment with sterile 20 mM Tris-HCl buffer (Sigma, St. Louis, MO) to detach contaminating germ cells. The results of islets, ECs and SCs identifications were provided in Figure S1 in Supplemental Materials.

### Endothelial Cells Coating Islets

One thousand IEQ islets were mixed together with 3.0 ×10^6^ ECs in 500 uL culture medium. ECs and islets were co-cultured in culture tube for 2 hours. After incubation the islets and ECs were transferred to 15 cm^2^ low adsorption treated petri dishes to prevent ECs from adherence to dishes. After 48 hours culture, ECs attached to islets and coated it.

### In vivo Experimental Groups and Islet Transplantation

Wistar rats were rendered diabetic by a single intraperitoneal injection of streptozotocin (STZ) at a dose of 220 mg/kg [22]. Diabetic Wistar rats were randomly assigned into four groups. Rats in the group A received non-treated islets transplantation under the left renal capsule as control group. For experimental groups, rats in group B received ECs-coated islets. In group C, Rats were injected with approximately 2×10^8^ Sertoli cells through the lateral tail vein before non-treated islets transplantation. ECs-coated islets were transplanted into rat in group D treated by Sertoli cells injection before transplantation. No other immunosuppressive protocols were used in each recipient. Blood glucose level less than 11.1 mmol/L on 2 consecutive days was defined as successful islet function.

### Graft Survival and Function

Blood glucose level less than 11.1 mmol/L on 2 consecutive days was defined as successful islet function. Blood glucose level more than 11.1 mmol/L on 2 consecutive days was defined as graft rejection. The mean survival time (MST) of islet graft in each group was recorded. Blood glucose levels and insulin concentrations were monitored to assess islet graft function.

### Immunohistological Analysis

The islet graft tissues of animals in each group were collected on the 7th day, which of animals in groups B, C, and D on the 14th day after transplantation in addition. Animals were killed by CO_2_, and islet grafts were retrieved from individual animals. After being fixed in 10% phosphate-buffered formalin overnight, islet grafts were embedded in paraffin and sectioned at 4.5 µm. The islet graft tissues of animals in each group were collected and stained with rabbit anti-rat insulin and vWF antibody respectively (1∶200 dilution; Santa Cruz Biotechnology, Inc., Santa Cruz, CA) using an immunoperoxidase technique. Coded slides were examined by light microscopy (IX71, Olympus Corporation, Tokyo, Japan). The mean microvessel densities (MVD) of islet graft were measured after vWF staining as described [17].

### Flow Cytometry

Peripheral blood samples were collected from the tail vein the day before transplantation, and 7, 14 days after transplantation, and peripheral blood lymphocytes were isolated by density gradient centrifugation using rat lymphocyte separating medium (Solarbio Science & Technology Co., Ltd., Beijing, China). The percentages of CD4+ and CD8+ T cells in peripheral blood were measured by flow cytometry as described in our previous study using phycoerythrin-conjugated anti-rat-CD4 (OX-35; IgG2α), fluorescein isothiocyanate-conjugated anti-rat-CD8 (OX-8; IgG1) antibodies and the isotype mAbs (mouse IgG2κ; all from BD Biosciences Pharmingen, San Jose, CA) [22].

### Cytokine Detection by ELISA

Blood sera were obtained from peripheral blood samples by centrifugation at 1000 rpm for 5 min the day before transplantation, and 7, 14 days after transplantation. Commercially available ELISA kits (IFN-γ, IL-2, and IL-4; Bender MedSystems, Vienna, Austria) were used to determine the concentrations of cytokines.

### Sertoli Cell Co-culture with ECs-coated Islets

At first, SCs were moved to six-well plates approximately 5×10^4^ cells/mL for adherent culture to be feeder layer. Then 300 IEQ islets or ECs-coated islets were co-cultured in the six-well plates separated from SCs layer by a 0.4 mm microporous membrane insert (Transwell, Corning, Inc., Lowell, MA, USA) with the RPMI 1640 culture medium which were changed every day.

### In Vitro Experimental Groups

The islets from one donor were divided into four experimental groups as following: A, untreated islets as control group; B, ECs-coated islets; C, islets co-cultured with SCs; D, ECs-coated islets co-cultured SCs. Cells in each group were incubated at 37°C 3% O_2_, 5% CO_2_ and 92% N_2_ atmosphere for 10 days which is equivalent to oxygen tension of sub renal capsule [23].

### In Vitro Function Test

In each group, the glucose-stimulated insulin secretion test was performed at 1,4,7 and 10 days after treatment. Islet-specific function was evaluated in a dynamic perfusion system as described in our previous study [24]. All experimental islets were challenged with two glucose concentrations (first in 1.67 mmol/L and then in 16.7 mmol/L). Fractions were collected and analyzed for insulin content using a commercial insulin enzymelinked immunosorbent assay (ELISA) kit, Mercodia Insulin ELISA (Mercodia, Sweden). Insulin Release Stimulation Index (SI) was calculated by relative value of insulin secretion during incubation with 16.7 mmol/L and 1.67 mmol/L glucose.

### Effect on ECs-coating of Sertoli Cells Co-lculture

The ECs-coated islets in each group were collected and were stained with rabbit anti-rat vWF antibody (1∶200 dilution; Santa Cruz Biotechnology, Inc., Santa Cruz, CA) and TRITC labeled sheep anti-rabbit second antibody (1∶500 dilution; Santa Cruz Biotechnology, Inc., Santa Cruz, CA). Coded slides were examined by fluorescence microscopy and relative fluorescence intensities were compared by Image Plus 6 software (MEDIA CYBERNETICS, USA) under 5 selected visual fields in each group.

### Western Blot Analysis

Islets or islet/ECs in four groups were collected by centrifugation after 24 hours culture. Selected proteins were extracted from cells and detected by Western blot as described in our previous study [16]. Cells were lysed in a buffer containing 60 mol/L Tris-HCl (pH 6.8), 1% sodium dodecyl sulfate, 10% glycerol, 0.5%-mercaptoethanol, 0.05% NP-40, and a protease inhibitor mixture (1∶100 dilutions). Equivalent amounts of protein from cells of each group were run on 6% to 12% sodium dodecyl sulfate polyacrylamide gels and electrically transferred to nitrocellulose filters. After nonspecific binding sites were blocked, the membranes were incubated for 2 h at room temperature with a rabbit polyclonal antibody against HSP-32 (1∶2000), Ki-67 (1∶1000), PDX-1 (1∶1000), Bax (1∶1000), Bcl-2 (1∶1000), Akt (1∶500), Phospho-Akt (1∶1000), ERK1/2 (1∶1000), phosphop-ERK1/2 (1∶1000), PLC-γ (1∶1000), phospho-PLC-γ (1∶1000), FAK (1∶1000), phospho-FAK (1∶1000), β-actin (1∶1000), NF-κB (1∶500) and KDR, 1∶0000 (all from Santa Cruz, CA) followed by a secondary peroxidase-linked anti-rabbit antibody (Tropix, PE Applied Biosystems, Freiburg, Germany;1∶10,000 dilution). Protein expression was visualized by means of luminal enhanced chemiluminescence (ECL plus, Amersham Pharmacia Biotech, Freiburg, Germany) and digitized with ChemiDocTM XRS System (Quantity One, Bio-Rad Laboratories GmbH, Munich, Germany). Signals were densitometrically assessed (Quantity One, Bio-Rad) and normalized to the b-actin signals as loading controls (mouse monoclonal anti-β-actin antibody, 1∶10,000; Sigma Chemical). The relative abundance of the different genes was calculated by the comparative _ΔΔ_Ct method [25].

### Mixed Leukocyte Reaction (MLR)

PKH-67 (Sigma, St. Louis, MO) labeled allogeneic naive splenocytes from adult male Wistar rats were prepared by tissue mincing and hypotonic lysis of red blood cells. Subsequently, the labeled splenocytes were co-cultured with islets of each group for 3 days. PKH-67 labeled splenocytes (1.0×10^6^) that were not co-cultured with islets were used as negative control group. Cell Quest PRO software (BD FACSCalibur, USA) was used to obtain the data and Modfit software was used to analyze the dynamic model of different cell subsets’ proliferation. The increment of different cell subsets in different activators was measured. Proliferation index (PI) was obtained directly from the Modfit software.

### ELISA Analysis of Cytokines

After a 48-hr incubation period, supernatants in each group were collected (n = 10). A commercial ELISA (rat VEGF-A, TGF-β, IL-1, IGF-1, EGF, and bFGF detection kit; Bender, Vienna, Austria) was used to determine the concentrations of cytokines.

### Statistical Analysis

The statistical significance of differences was determined using one-way analysis of variance tests. Statistical analyses were performed using SPSS version 13.0 (SPSS Inc., Chicago, IL). Differences between the blood glucose and insulin concentration at ten times under the four conditions were analyzed by multivariate analysis using SAS 9.0 (SAS Inc., Chicago, IL). A P-value less than 0.05 were considered statistically significant.

## Results

### ECs Coating and SCs Infusion Strategy Improves Islet Graft Survival and Function in Renal Capsule

**Figure 1:**
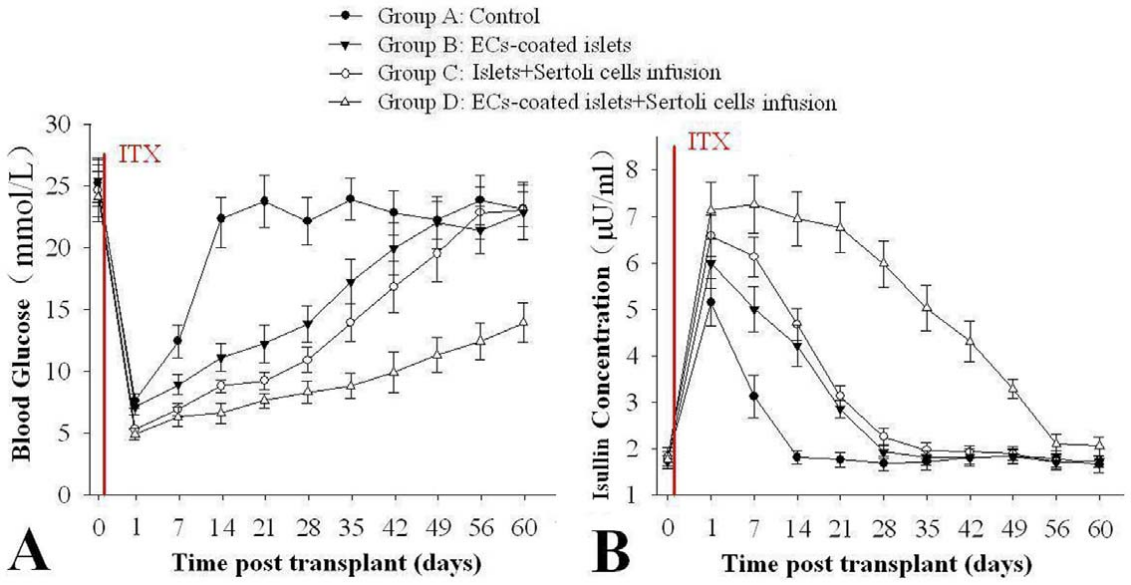
ECs-coating and SCs infusion improve of islet grafts function in vivo after transplantation. Compared with the control group (group A), ECs-coated islets and Sertoli cells before transplantation demonstrated enhanced glucose tolerance ability in blood glucose levels and increase in blood insulin concentration (P<0.05 vs. each other, n = 10).

Compared group A as control (5.2±1.14 days), Treatments in group B, C, and D all showed some potential to prolong the survival of graft (Table 1). The MST of groups B, C, and D had significantly longer MSTs of 15.2±2.27 days, 29.8±1.27 days (P<0.05), and 57.6±3.91 days (P<0.01) than that of control group. The combined strategy of ECs coating and SCs infusion in group D markedly promoted graft function, and they had the longest MST of graft. In addition, the MST of group B was significantly shorter than that of group C (P<0.05). After glucose administration, however, the group that D was transplanted with ECs-coated islets and SCs infusion demonstrated significantly lower levels of glucose than the other groups from 0 to 49 days post transplantation followed by group B, C and A (P<0.05, Fig. 1A). The changes of insulin concentrations level were is inversely proportional to glucose levels. Rats of recipients in group D had the highest insulin followed by group B, C and A from 0 to 49 days post transplantation (P<0.05, Figure 1B).

**Table 1:** ECs coating and SCs infusion prolong islet allograft survival in diabetic Wistar rat recipients.

Group	n	Treatment	Graft survival (days)	Mean survival Time (days)
A	10	Untreated islets	6,5,4,7,5,4,5,7,4,5	5.2±1.14
B	10	ECs-coated islets	9,13,30,25,14,11,10,12,9,19	15.2±2.27*
C	10	Sertoli cells infusion	28,33,39,27,31,25,29,30,30,26	29.8±1.27*^#^
D	10	ECs-coated islets +Sertoli cells infusion	47,55,68,81,40,50,70,49,56,60	57.6±3.91*^#^∧

Groups B, C, and D had significantly longer MSTs compared with that of control group (P<0.05). The combined strategy in group D had a significant longer MST than those of groups B and C (P<0.05). In addition, the MST of group C was significantly shorter than that of group D (P<0.05). * P<0.05 vs. group A, # P<0.05 vs. group B, ∧ P<0.05 vs. group C.

SI: A, 8.90±0.24; B, 6.34±0.19; C, 2.21±0.15; D, 2.47±0.18.

### Combined Strategy Promotes Insulin Release and Vascularization of Islet Graft

**Figure 2:**
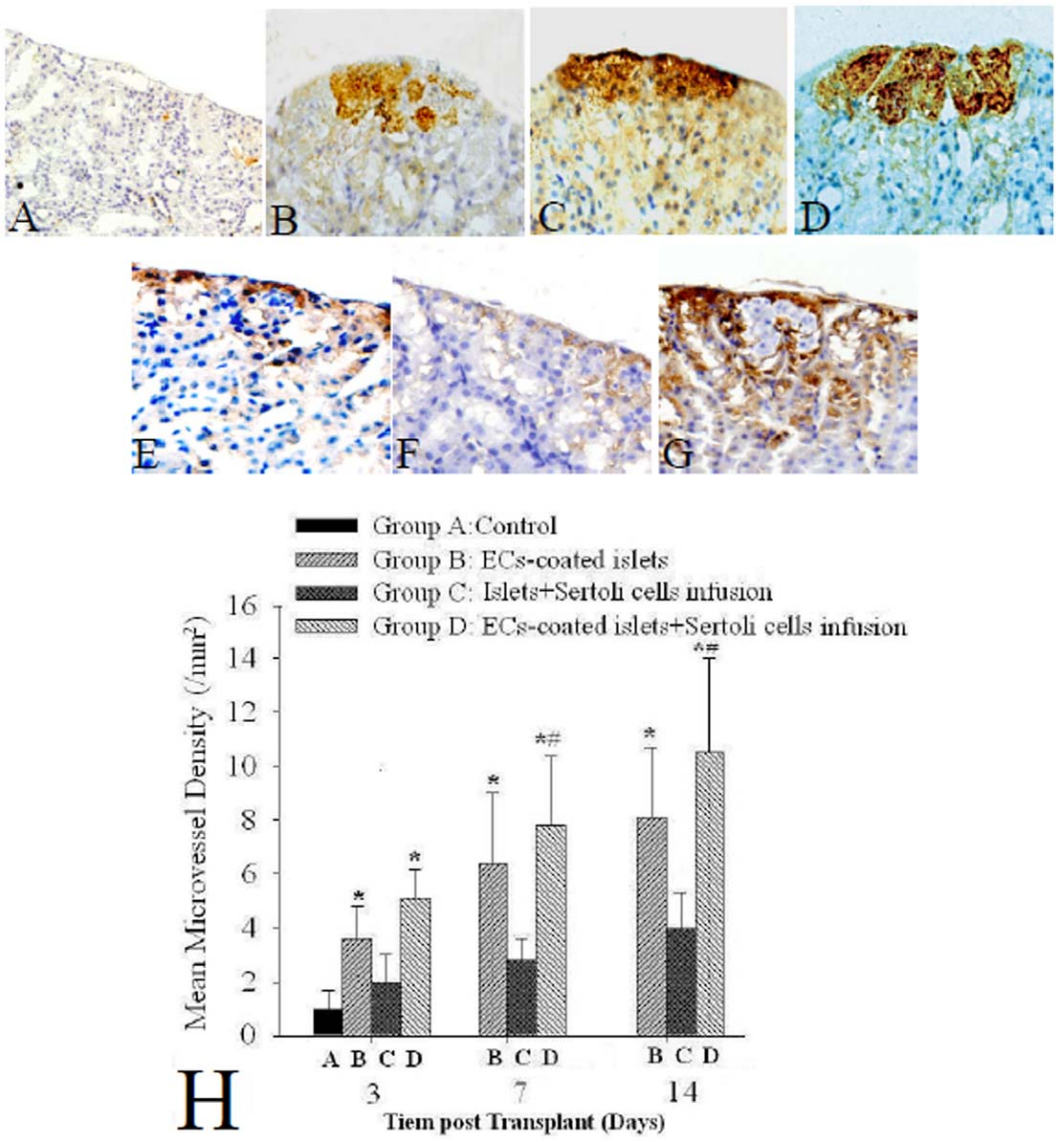
Detection of insulin, vWF and mean microvessel densities (MVD) in grafts transplanted into renal subcapsule by immunohistochemistry. (A) In control group (islets only), the expression of insulin became undetectable 7 days after transplantation (magnification×100); (B, C) The grafts in groups B (ECs-coated islets) and C (SCs co-cultured islets), however, still had detectable islet graft secreting insulin under renal capsule 14 days after transplantation. (magnification×200). (D) In group D (ECs coated and SCs co-cultured islets), large number of insulin-positive grafts were observed 14 days after transplantation (magnification×200). (E). Quite a lot vWF positive ECs around islet were detected in group B 14 days after transplantation (magnification×200). (F) Only slight vWF positive ECs around islet in group C 14 days after transplantation (magnification×200). (G). There were the most vWF positive ECs around islets in group D 14 days after transplantation (magnification×200). (H) The MVD in grafts of groups B and B were significantly higher than those in groups A and C from 3 to 14 days after transplantation. The MVD in group D was significantly higher than that in group B at 7 and 14 days post-transplantation respectively. * P<0.05 vs. group A and C, #P<0.05 vs. group B, n = 10.

Immunohistochemistry showed that insulin secreting by islet grafts in control group were not detectable under renal capsule 7 days after transplantation. In groups B, C, and D, however, insulin expressions of islet grafts were still detected under renal capsule 14 days post-transplantation. The islet graft in group D had the highest insulin expression intensity (Figure 2D), followed by that in group C and B (Figure 2B and C). Quite a lot ECs coating islets were detected by vWF stainging in graft of group B (Figure 2E) 14 days after transplantation. Fewer vWF positive ECs around islets in group C (Figure 2F) at the same time. While in group D, islet grafts were surrounded by most vWF-positive ECs (Figure 2G) 14 days post-transplantation. The MVD in grafts of groups B and D were significantly higher than those in groups A and C from 3 to 14 days after transplantation. The MVD in group D was significantly higher than that in group B at 7 and 14 days post-transplantation respectively. (P<0.05, Figure 2H). The blood vessel formation and area of grafts were increased by ECs-coated compared with the non ECs-coated grafts. SCs co-culture could further improve the blood microvessel of grafts.

### Sertoli Cells Infusion Inhibits Lymphocyte Activation and Inflammatory Factor of Recipient by Islet and ECs

**Figure 3:**
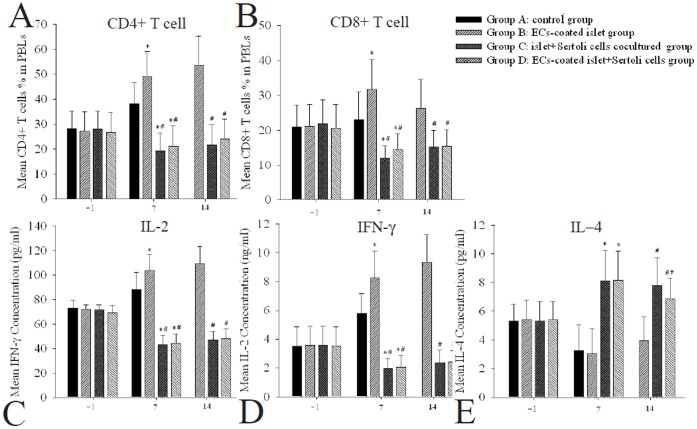
The changes of lymphocytes and levels of cytokines in peripheral blood of rats after islet transplantation. (A, B) Seven and 14 days after transplantation, ECs coated islet grafts actived CD4+ and CD8+ T cells and SCs infusion could inhibit lymphocyte activation. (C, D) and Seven and 14 days after transplantation, ECs coated islet grafts group had significantly higher IL-2 and IFN-γ levels than those in other groups. (E) Seven and 14 days after transplantation, IL-4 levels in groups C and D were higher than that in groups A and B. IL-4 level in group D was significantly lower than that in group C. * P<0.05 vs. group B, # P<0.05 vs. group A, †P<0.05 vs. group C, n = 10.

There were no significant differences in percentage of lymphocyte subpopulation and cytokines of the recipient rats (Figure 3).

As shown in Figure 3A and B, transplanted islet grafts actived the CD4+ and CD8+ T cells in peripheral blood of the rats and enhanced by ECs coating (group A and B). While SCs infusion inhibited lymphocyte activation stimulated by islet grafts which could still keep even the presence of ECs (group C and D).

As shown in Figure 3C and D, the changes of IL-2 and IFN-γ had similar trends as lymphocyte. Islet or ECs coated islet grafts caused the increase of IL-2 and IFN-γ SCs infusion decreased the release of IL-2 and IFN-γ . In contrast, IL-4 showed significantly higher level in rats with SCs infusion than that without infusion and not affected by ECs coating (Figure 3E).

### Insulin Secretion Following Glucose Stimulation

**Figure 4:**
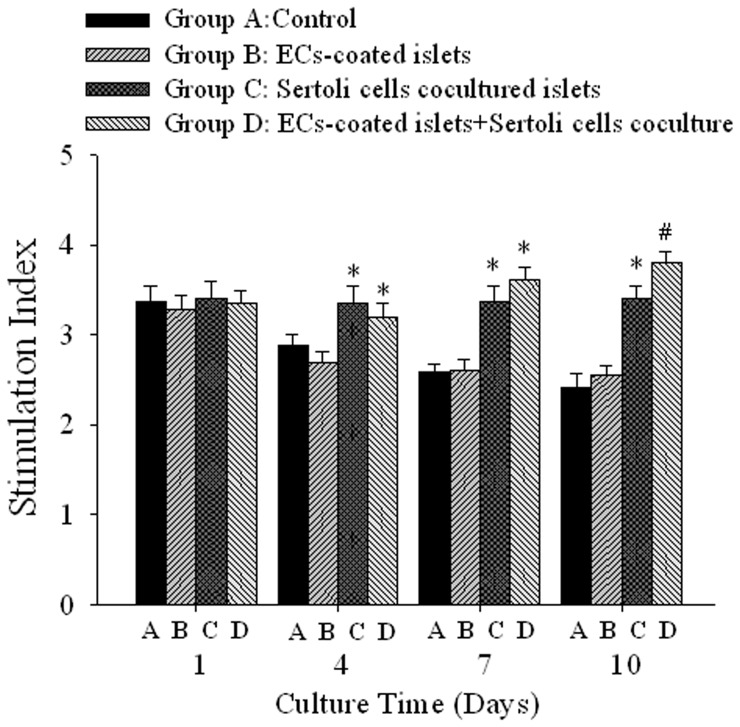
Combined strategy of ECs coating and SCs coculture improves insulin release stimulation index ranther than single treatment. The SI had no difference between control and ECs coated islets during 10 days culture. Islet or ECs coated islets had higher SI with SCs coculture. In two SCs co-culture groups the SI of ECs coated islets was superior to that of islets. * P<0.05 vs. group A and B, # P<0.05 vs. group C, n = 10.

As shown in Figure 4, there were no significant differences of SI between each group after 1 day culture. Groups C and D began a significantly higher SI than those in groups A and B from 4 days culture and kept similar statement at 10 days culture (P<0.05). There was a significant difference in SI levels between groups C and D after 10 days culture (P<0.05). The detailed values of insulin and SI were provided in Table S1 of supplemental materials.

### Improvement of ECs-coating Islet by Sertoli Cells Coculture

**Figure 5:**
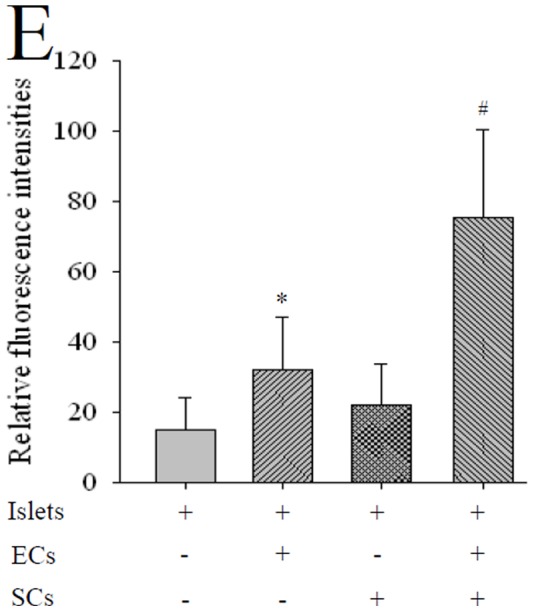
Improvement of SCs co-culture to the effect of ECs coating islets. Immunofluorescence showed that nearly no vWF positive ECs were detected around islets in control group (islets only) after 7 days culture. ECs expressing vWF were still detectable in ECs coating and SCs coculture groups by 7 days culture. The islets in combined strategy group had the most vWF-positive ECs around it after 7 days co-culture. * P<0.05 vs. islet and SCs coculture, # P<0.05 vs. ECs coating, n = 10.

vWF-stained ECs appeared red fluorescence (Figure S2 in Supplemental Materials). Immunofluorescence showed that the rats in group A nearly did not have detectable around islet 7 days after culture. ECs were still detectable 7 days after culture in group B and C. The islet in ECs-coated and SCs coculture group had the most vWF-positive ECs around it after 7 days culture and the fluorescence intensity of which was stronger than those of other groups (P<0.05, Figure 5).

### Combined Strategy brings Alteration of Cell Survival, Apoptosis, and Angiogenesis-related Signaling

**Figure 6:**
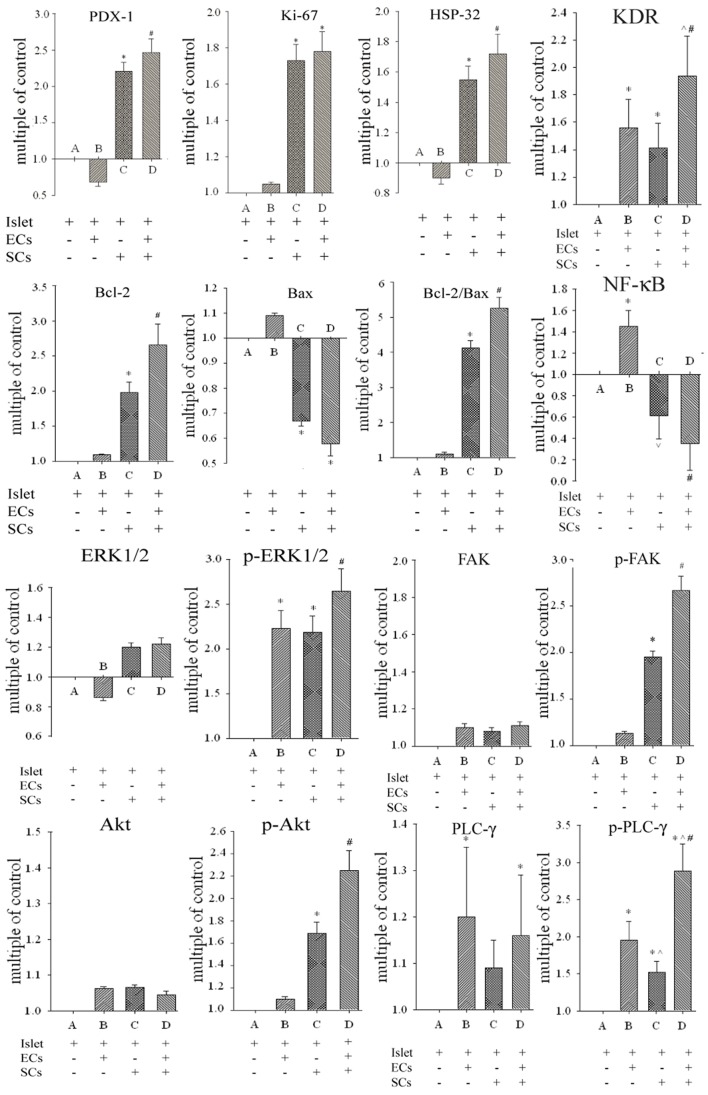
The effects of different treatments on the expressions of survival, proliferation, and angiogenesis related signal proteins. Western blot analysis investigated the control of survival, proliferation, and angiogenesis related signal induction of ECs coated and SCs co-cultured islets. Single treatment of ECs coating or SCs co-culture both had effect on expressions of some signal proteins. Combined strategy better improved survival, proliferation, and angiogenesis related signal proteins expression. Signals were densitometrically assessed and normalized to the β-actin signals as loading controls. * P<0.05 vs. group A, ∧ P<0.05 vs. group B, # P<0.05 vs. group C, n = 10.

As shown in Figure 6, expression of PDX-1, Ki-67 and HSP-32 were increased by in group C (SCs co-culture).(P<0.05). Combined strategy of ECs coating and SCs co-culture in group D further enhanced the expression of PDX-1, HSP-32 (P<0.05). There was an evident impact to expression levels of Bax and Bcl-2 in group (P<0.05) and the impact became more significant in group D (P<0.05). The expression of ERK-1/2, FAK and Akt had no obviously changes between four groups. The expression of p-ERK1/2 was both altered in groups B and C and further increased in group D (P<0.05). The p-FAK and p-Akt expressions increased in groups C and D and there was a significant difference between them (P<0.05). Our analysis demonstrated an increased expression of PLC-γ in groups B and D which had no difference between them. While group D had a strongest expression of p-PLC-γ followed by groups B and C (P<0.05). Expression of KDR were all increased in groups B, C and D and the expression in group D was significantly stronger than those in other two groups (P<0.05). NF-κB were obviously increased in group B and inhibited in groups C and D (P<0.05). The expression of NF-κB was lower in group D than that in group C (P<0.05).

### The Immunogenicity of ECs was Contained by Co-culturing with Sertoli Cells

**Figure 7:**
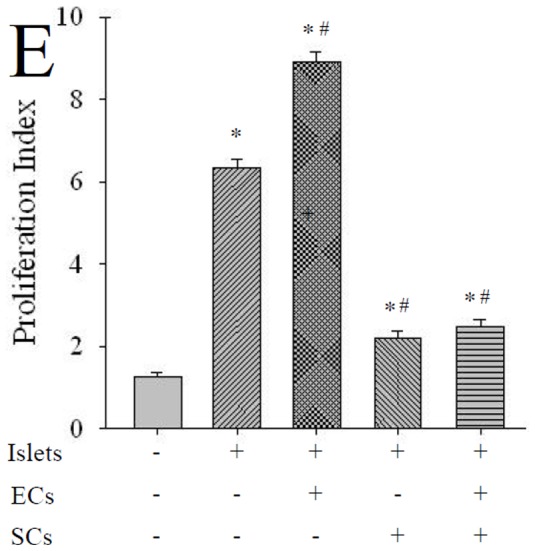
SCs coculture inhibits proliferation of lymphocyte in MLR. The PI in different group. ECs coating could significantly stimulate prolife ration of spleen cells compared with the control group. The PI was significantly reduced by co-culture with SCs whether ECs coated or not. * P<0.05 vs. group negative control, # P<0.05 vs. islet group, n = 10.

Significant proliferation was observed in the mononuclear cells co-cultured with each group of islet cells (Figure 7). ECs coating could significantly stimulate proliferation of spleen cells compared with the control group A (P<0.05). The PI was significantly reduced by SCs co-culture no matter ECs coated or not. The detailed figure and PI were provided in Figure S3 in Supplemental Materials.

### Concentrations of Cytokines in Culture Supernatants

**Figure 8:**
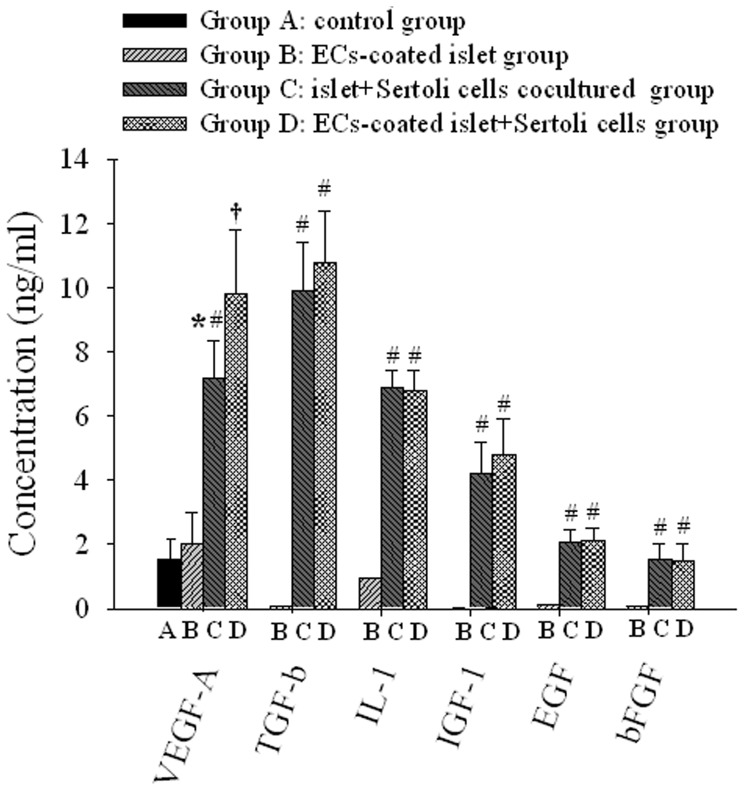
SCs secretes some cytokines and also improves VEGF-A secreted by islets. The EC-coating and SCs co-culture improved islet quality and induced related cell signals, despite the lack of any direct contact between islets and SCs. This implies that improved islet quality is linked to SCs-derived molecules such as cytokines and growth factors. To identify the secreted soluble factors, cells in each group were cultured for 48 hr, after which the medium supernatant was harvested to determine the identity and amount of soluble factors using ELISA. VEGF-A and transforming growth factor TGF-β, IL-1, IGF-1, and basic fibroblast growth factor (bFGF) were detected, n = 10.

We performed an ELISA to identify some soluble factors of VEGF-A, TGF-β, IL-1, IGF-1 EGF, and bFGF (Figure 8). Except for VEGF-A, other cytokines were all not detected in medium of group A. Among these factors, IL-1, and IGF-1 were detected at significant concentrations in supernatants containing SCs (group C and D) which had no significantly difference. In particular, a high concentration of TGF-β was detected in group C and D. EGF and bFGF were also detected in group C and D, but at very low concentrations still no difference was seen between them. The VEGF-A concentrations in medium of group B, C and D were all significantly higher than that in group A (P<0.05). Supernatants in of ECs coating and SCs co-culture group D had an highest concentrations of VEGF-A followed in group C and D which also had difference between each other (P<0.05). The detailed concentrations were provided in Table S2 in Supplemental Materials.

## Discussion

Although intensive investigations have been focused on IBMIR inhibitory effect of ECs-coating islets [10], little attention is paid on its role in vascularization which represents a potential improvement for the survival of islet grafts. However, the effect of ECs-coated islets alone on the promotion of vascularization and survival of islets is still limited. ECs-coating can also affect the exchange of nutrients and oxygen in islets as well as insulin secretion [24], [26]. Under the environment of hypoxia and deficient nutrients, the fast apoptosis of islets can result in the failure of coating [27]. In previous studies, the transfection of angiogenic factors has been applied for promoting the proliferation of ECs in islets and improving vascularization speed [28]. However, the application of this method is also limited due to the low transfection efficiency of adenovirus vector and cell damage from gene modification. Islet transplantation received combinatorial treatment of ECs coating and SCs infusion of can avoid cell damage from gene modification during the environment of islet re-construction from natural sources. In the present study, islet transplantation with combinatorial treatment facilitates the longest MST and the best regulation of blood glucose which demonstrates this combinatorial strategy significantly improves the survival of islets and enhances the expression of insulin [16].

Our study has demonstrated that combinatorial strategy is a novel method for revascularization and rejection protection as well as survival improvement of islet grafts. Histological analysis has revealed an increased insulin expression and angiogenesis in islet through ECs coating and SCs infusion, which is consistent with previous reports that well-developed vascular structure improves insulin secretion through interaction between β-cells and ECs [29]. However, in vivo evidence has also demonstrated the difference in improvement of ECs coating and enhancement of β-cell function support the hypothesis that increased angiogenesis in ECs-coated and SCs-co-cultured islet grafts can be induced by a trophic factor related to SCs, which then induces the signals involved in β-cell survival and extra ECs revascularization.

We have established the system of ECs coating and SCs co-culture to explore the mechanism of this combinatorial strategy. In previous studies, the co-culture of islets and SCs was conducted in a simple way due to the presence of physical interactions [30]. The SCs infusion may not communicate with ECs-coating islet grafts during transplantation in vivo. Therefore, in the present study, we have exploited the special wells with microporous membrane between ECs-coating islets and SCs, thereby blocking their physical interactions to avoid the influence of SCs on ECs and islets. Our co-culture method will contribute to the nutritional effect of SCs on islets and ECs. Although ECs-coating can improve the number of ECs in islets, no obvious improvement on the function and microvascular vessels of islets is observed. Without direct interaction, the nutritional factors secreted by co-cultured SCs improve the function of islets, but ECs coating islets alone do not reveal an obvious increase in the function of islets. The combinatorial application of both SCs and ECs has a synergistic effect on improving the survival and function of islets, maintaining better morphology of islets, and providing more attachment of ECs on the surface of islets and enough time for its’ proliferation, as well as further enhancing ECs population on the surface of islets and promoting angiogenesis.

The expression and phosphorylation of various signaling proteins can provide the insight into underlying events. The combinatorial strategy of ECs-coating islets and SCs co-culture can significantly improve the expression of PDX-1, Ki-67, HSP-32 and Bcl-2, and simultaneously inhibit the expression of Bax. PDX-1 can increase the regeneration and proliferation of β cells and HSP-32 has a cytoprotective effect on islets as well as the suppressive function on inflammatory reactions and oxidative stress [30], [31]. In addition, Bcl-2 and Bax are known as anti-apoptotic and pro-apoptotic signaling molecules in cells [32]. SCs co-culture rather than ECs-coating can regulate the expression of signaling molecules in islets, promote the regeneration and repairing of β-cells, and enhance the tolerance of hypoxia and anti-apoptotic capability, thus promoting the survival and function of islets. No obviously increased expressions of Ki-67 and Bax are observed when compared with SCs co-culture alone, which may be due to the effect of combinatorial strategy on insulin secretion of islets, rather than the proliferation.

Akt and ERK1/2 play a crucial role in the survival and proliferation of cells [33], [34]. Akt and ERK1/2 not only affect the survival of islets, but also affect the proliferation of ECs. Similarly, our previous studies have also revealed that the SCs co-culture significantly reduced the expression of caspase family members in islets and inhibit apoptosis [16]. In the present study, the anti-apoptotic function is activated by upstream Akt. The combinatorial strategy has obvious up-regulations of p-Akt and p-ERK1/2 when compared with single treatment. These results suggest that the combinatorial strategy can obviously enhance the resistance of hypoxia and apoptosis through activating Akt and ERK1/2 pathways and improving islet function, which can provide excellent fundament and efficiency of ECs-coating. As for the downstream signaling molecule such as NF-κB, ECs-coating significantly enhances the expression of NF-κB; on the other hand, the combinatorial strategy significantly inhibits the expression of NF-κB. Under the hypoxia environment, the up-regulation of NF-κB can attenuate the expression of anti-hypoxia and anti-apoptotic signaling molecules, thus reducing the survival of islets so that the reduced expression of NF-κB can prolong the survival of islets under the hypoxia environment [35]. During the combinatorial treatment in the present study, the inhibition expression of NF-κB can increase anti-hypoxia and anti-apoptotic signaling molecules. However, the independent NF-κB pathways for affecting the expression of signaling molecules are still unclear and need to be further explored.

Our current study has also confirmed that the combinatorial strategy can promote vascularization of islets through not only improving the survival of islets but also activing angiogenesis- or revascularization-related proteins and cytokines including FAK, PLC-γ, VEGFR2 (KDR) and VEGF-A which don’t express in islets themselves [36]. The phosphorylation of FAK is an upstream signal molecule related to VEGF-A-mediated migration of ECs. The phosphorylation of PLC-γ is an upstream signal molecule related to VEGF-A-mediated proliferation of ECs [37]. The improvement of islet function can increase the secretion of VEGF in hypoxia condition [38]. Our study has shown that islets can secret slight VEGF-A during the alone culture. The VEGF-A can be increased by the co-culture of SCs rather than ECs coating. After coating of ECs, both PLC-γ and p-PLC-γ can reveal a simultaneous increase in expression level. In addition, the expression of KDR also reveals an obvious enhancement; in contrast, the expression level of VEGF-A does not exhibit an obvious increase, suggesting that the enhanced expressions of PLC-γ and KDR are due to the increased number of ECs on the surface of islets but not to increased activity of these molecules. The co-culture of SCs can significantly increase the expression of p-PLC-γ, p-FAK and KDR on the surface of islets but the expression levels are lower than those in ECs-coated islets which may due to there is less ECs in SCs co-cultured islets. But high expression of p-FAK demonstrates increase migration and adhesion functions of ECs which may be associated to high concentration of VEGF-A in SCs co-culture medium. The combinatorial strategy may further improve VEGF-A expression in islets and activate signal molecules such as KDR, p-PLC-γ and p-FAK, thus promoting the angiogenesis of islets.

Due to the dual stimulation of ECs and islets from allogeneic sources, the significantly increased proportion of lymphocytes in receptors and expression levels of inflammatory factors can result in rejection response. Although the expression level of IL-4 does not reveal an obvious increase, its roles in the rejection response are still controversial [39]. The SCs infusion can inhibit lymphocytes and inflammatory factors; however, no reduction in function is due to the appearance of ECs, thereby maintaining the immunosuppressive effect during vascularization process. Our study has demonstrated that alone cultured islets can stimulate rapid proliferation of spleen lymphocytes, which further promote ECs-coating. Our data have clearly described that SCs can reduce the immunogenicity of co-cultured islets. The lymphocytes can not be further stimulated by ECs-coated islets in the presence of SCs co-culture because TGF-β1 secreted by SCs has a broad inhibitory effect on lymphocytes [22]. This may also be the reason for no obvious increase of lymphocyte stimulation index when ECs-coated islets are co-cultured with SCs. The effect of Fas-L expressed on surface of SCs on the induction of lymphocyte apoptosis is not observed because lymphocytes and SCs had no physical interaction each other. Therefore, the effect of lymphocyte inhibition may be even stronger in vivo due to the presence of Fas-L in SCs.

In addition, SCs secrete many types of active proteins as a nutrition source, which can improve the survival and function of islets and promote the vascularization of ECs-coated islet grafts. ELISA results have showed that TGF- β, IL-1, IGF-1, EGF and bFGF cannot be detected in culture supernatant from the control group at any time points. Conversely, these cytokines can be detected in SCs co-culture supernatant. These cytokines have different effects on islets and ECs. Previous studies have reported that IGF-1 can increase the expression of VEGF and EGF, and bFGF which can promote proliferation and migration of ECs [40], [41]. In addition, TGF-β can not only up-regulate the expression of HSP-32 for enhancing islet grafts’ resistance to hypoxia, but also exert immunosuppressive effects [22], [42].

In summary, we have applied a unique system of ECs coating and SCs co-culture to facilitate islet vascularization in vitro, which can significantly improve the function of islets and inhibit the activation of lymphocytes. In addition, the survival of islet grafts can be effectively prolonged through ECs coating and SCs infusion to affect vascularization promotion and immunological rejection inhibition.

## Supporting Information

Figure S1 **Islets, endothelial cells and Sertoli cells after separation and purification.** (A) Optical microscopic observation of DTZ-stained islets. The islets appeared as scarlet, less acinar, and highly pure (magnification× 100). (B) Fluorescence microscopic observation of acridine orangepropidium iodide–stained islets. Most islets showed green fluorescence, indicating good activity (magnification×100). (C) Immunocytochemistry detected vimentin expression (magnification×100). (D) Western-blotting detected Sox-9 expression in cultured Sertoli cells after isolation. (E) Immunofluorescence detected vWF expression (magnification×100). (F) ECs were observed by TEM. White arrows point to Weibel-Palade bodies which was a characteristic of ECs. (magnification×10000).
(TIF)

Figure S2 **Detection of vWF in islets after culture by immunofluorescence 7 days after culture.** (A) In control group, the expression of vWF was nearly undetectable 7 days after culture; (B) In the ECs-coated group, small amount of ECs around islets, which is significantly less than those of groups D; (C)In Sertoli cells co-cultured group, there were nearly no ECs around islets. (D) In ECs-coated islets and Sertoli cells coculture group, large number of vWF-positive ECs around islet. Magnification×200.
(TIF)

Figure S3 **MLR and proliferation index in each group.** Lymphocyte mixed with (A)islets; (B)ECs-coated islets; (C)Islets co-cultured with Sertoli cells. (D)ECs-coated islets co-cultured with Sertoli cells. Magnification×100.
(TIF)

Table S1 **Insulin release and stimulation index (SI) by glucose-stimulation test in four groups at different culture time.**
(DOC)

Table S2 **Cytokines secretion profiles in culture media in each group.**
(DOC)

## References

[1] Shapiro AM, Lakey JR, Paty BW, Senior PA, Bigam DL, et al (2005) Strategic opportunities in clinical islet transplantation. Transplantation 79: 1304–1307. 15912095 10.1097/01.tp.0000157300.53976.2a

[2] Hering BJ (2005) Achieving and maintaining insulin independence in human islet transplant recipients. Transplantation 79: 1296–1297. 15912092 10.1097/01.tp.0000157321.55375.86

[4] Ichii H, Sakuma Y, Pileggi A, Fraker C, Alvarez A, et al (2007) Shipment of human islets for transplantation. Am J Transplant 7: 1010–1020. 17391141 10.1111/j.1600-6143.2006.01687.x

[5] Stagner JI, Samols E (1990) The induction of capillary bed development by endothelial-cell growth-factor before islet transplantation may prevent islet ischemia. Transplant Proc 22: 824–828. 1691556

[6] Fiorina P, Folli F, Maffi P, Placidi C, Venturini M, et al (2003) Islet transplantation improves vascular diabetic complications in patients with diabetes who underwent kidney transplantation:a comparison between kidney-pancreas and kidney-alone transplantation. Transplantation 75: 1296–1301. 12717219 10.1097/01.TP.0000061788.32639.D9

[7] Menger MD, Jaeger S, Walter P, Feifel G, Hammersen F, et al (1989) Angiogenesis and hemodynamics of microvasculature of transplanted islets of langerhans. Diabetes 38: 199–201. 2463196 10.2337/diab.38.1.s199

[8] Linn T, Schneider K, Hammes HP, Preissner KT, Brandhorst H, et al (2003) Angiogenic capacity of endothelial cells in islets of langerhans. FASEB J 17: 881–883. 12670881 10.1096/fj.02-0615fje

[9] Pan XM, Xue WJ, Li Y, Feng XS, Tian XH, et al (2011) Islet graft survival and function: concomitant culture and transplantation with vascular endothelial cells in diabetic rats. Transplantation 92: 1208–1214. 22067310 10.1097/TP.0b013e3182356ca7

[10] Song HJ, Xue WJ, Li Y, Tian XH, Ding XM, et al (2010) Prolongation of islet graft survival using concomitant transplantation of islets and vascular endothelial cells in diabetic rats. Transplant proc 42: 2662–2665. 20832565 10.1016/j.transproceed.2010.06.003

[11] Johansson U, Elgue G, Nilsson B, Korsgren O (2005) Composite islet-endothelial cell grafts: a novel approach to counteract innate immunity in islet transplantation. Am J Transplant 5: 2632–2639. 16212622 10.1111/j.1600-6143.2005.01076.x

[12] Vajkoczy P, Menger MD, Simpson E, Messmer K (1995) Angiogenesis and vascularization of murine pancreatic islet isografts. Transplantation 60: 123–127. 7542814

[13] Li Y, Yan H, Xue WJ, Tian PX, Ding XM, et al (2009) Allograft rejection-related gene expression in the endothelial cells of renal transplantation recipients after cytomegalovirus infection, J Zhejiang Univ Sci B. 10: 820–828. 10.1631/jzus.B0920115 PMC2772886 19882756

[14] Tajik N, Salari F, Ghods AJ, Hajilooi M, Radjabzadeh MF, et al (2008) Association between recipient ICAM-1 K469 allele and renal allograft acute rejection. Int J Immunoqenet 35: 9–13. 10.1111/j.1744-313X.2007.00727.x 18186794

[15] Griswold MD (1998) The central role of Sertoli cells in spermatogenesis. Semin Cell Dev Biol 9: 411–416. 9813187 10.1006/scdb.1998.0203

[16] Rodriguez AI, Willing AE, Saporta S, Cameron DF, Sanberg PR (2003) Effects of Sertoli cell transplants in a 3-nitropropionic acid model of early Huntington’s disease; a preliminary study. Neurotox Res 5: 443–450. 14715448 10.1007/BF03033174

[17] Li Y, Xue WJ, Tian XH, Ding XM, Tian PX, et al (2011) Improved survival and function of rat cryopreserved islets by co-culture with Sertoli cells. Artifi Organ 35: 634–644. 10.1111/j.1525-1594.2010.01155.x 21371055

[18] Sakata N, Chan NK, Chrisler J, Obenaus A, Hathout E (2010) Bone marrow cell cotransplantation with islets improves their vascularization and function. Transplantation 89: 686–693. 20101199 10.1097/TP.0b013e3181cb3e8d PMC2844476

[19] Fan P, He L, Pu D, Lv XH, Zhou WX, et al (2011) Testicular Sertoli cells influence the proliferation and immunogenicity of co-cultured endothelial cells. Biochem Biophys Res Commun 404: 829–833. 21172306 10.1016/j.bbrc.2010.12.068

[20] Skinner MK (1991) Cell-cell interaction in the testis. Endocr Rev 12: 45–77. 2026122 10.1210/edrv-12-1-45

[21] Ahn YO, Lee JC, Sung MW, Heo DS (2009) Presence of membrane-bound TGF-beta 1 and its regulation by IL-2-activated immune cell-derived IFN-gamma in head and neck squamous cell carcinoma cell lines. J Immunol 182: 6114–6120. 19414763 10.4049/jimmunol.0803725

[22] Muruve DA, Nicolson AG, Manfro RC, Strom TB, Sukhatme VP, et al (1997) Adenovirus-mediated expression of Fas ligand induces hepatic apoptosis after systemic administration and apoptosis of ex vivo-infected pancreatic islet allografts and isografts. Hum Gene Ther 8: 955–963. 9195218 10.1089/hum.1997.8.8-955

[23] Li Y, Xue WJ, Tian XH, Feng XS, Ding XM, et al (2010) Study on systemic immune tolerance induction in rat islet transplantation by intravenous infusion of sertoli cells. Transplantation 2010 89: 1430–1437. 10.1097/TP.0b013e3181da607e 20463639

[24] Kilani RT, Mackova M, Davidge ST, Guilbert LJ (2003) Effect of oxygen levels in villous trophoblast apoptosis. Placenta 24: 826–834. 13129679 10.1016/s0143-4004(03)00129-2

[25] Li ZL, Xue WJ, Tian PX, Liu H, Feng XS, et al (2008) Establishment of a rat islets model coated with human umbilical vein endothelial cells coexpressing sCD40L-Ig and CTLA4-Ig. Chin J Cell Mol Immunol 24: 1147–1149. 19068196

[26] Calzado MA, de la Vega L, Moller A, Bowtell DD, Schmitz ML (2009) An inducible autoregulatory loop between HIPK2 and Siah2 at the apex of the hypoxic response. Nat Cell Biol 11: 85–91. 19043406 10.1038/ncb1816

[27] Robertson RP (2004) Islet transplantation as a treatment for diabetes-A work in progress. N Engl J Med 350: 694. 14960745 10.1056/NEJMra032425

[28] Ahlgren U, Jonsson J, Jonsson L, Simu K, Edlund H (1998) β-Cell-specific inactivation of the mouse Ipf1/Pdx1 gene results in loss of the β-cell phenotype and maturity onset diabetes. Genes Dev 12: 1763–1768. 9637677 10.1101/gad.12.12.1763 PMC316911

[29] Zhang N, Richter A, Suriawinata J, Harbaran S, Altomonte J, et al (2004) Elevated vascular endothelial growth factor production in islets improves islet graft vascularization. Diabetes 53: 963–970. 15047611 10.2337/diabetes.53.4.963

[30] Johansson M, Mattsson G, Andersson A, Jansson L, Carlsson PO (2006) Islet endothelial cells and pancreatic beta-cell proliferation: Studies in vitro and during pregnancy in adult rats. Endocrinology 147: 2315–2324. 16439446 10.1210/en.2005-0997

[31] McKinnon CM, Docherty K (2001) Pancreatic duodenal homeobox-1, PDX-1, a major regulator of beta cell identity and function. Diabetologia 44: 1203. 11692168 10.1007/s001250100628

[32] Lee DY, Lee S, Nam JH, Byun Y (2006) Minimization of immunosuppressive therapy after islet transplantation: Combined action of heme oxygenase-1 and PEGylation to islet. Am J Transplant 6: 1820–1828. 16780547 10.1111/j.1600-6143.2006.01414.x

[33] Plesner A, Liston P, Tan R, Korneluk RG, Verchere CB (2005) The X-linked inhibitor of apoptosis protein enhances survival of murine islet allografts. Diabetes 54: 2533–2540. 16123340 10.2337/diabetes.54.9.2533

[34] Meng ZX, Sun JX, Ling JJ, Lv JH, Zhu DY, et al (2006) Prostaglandin E2 regulates Foxo activity via the Akt pathway: Implications for pancreatic islet beta cell dysfunction. Diabetologia 49: 2959. 17033838 10.1007/s00125-006-0447-5

[35] Dalle S, Longuet C, Costes S, Broca C, Faruque O, et al (2004) Glucagon promotes cAMP-response element-binding protein phosphorylation via activation of ERK1/2 in MIN6 cell line and isolated islets of langerhans. J Biol Chem 279: 20345–20355. 14988413 10.1074/jbc.M312483200

[36] Chen C, Moreno R, Samikannu B, Bretzel RG, Schmitz ML, et al (2011) Improved intraportal islet transplantation outcome by systemic IKK-beta inhibition: NF-κB activity in pancreatic islets depends on oxygen availability. Am J Transplant 11: 215–224. 21219576 10.1111/j.1600-6143.2010.03390.x

[37] Negi S, Jetha A, Aikin R, Hasilo C, Sladek R, et al (2012) Analysis of beta-cell gene expression reveals inflammatory signaling and evidence of dedifferentiation following human islet isolation and culture. PloS One 7: e30415. 22299040 10.1371/journal.pone.0030415 PMC3267725

[38] Matsumoto T, Mugishima H (2006) Signal transduction via vascular endothelial growth factor (VEGF) receptors and their roles in atherogenesis. J Atheroscler Thromb 13: 130. 16835467 10.5551/jat.13.130

[39] Johansson U, Olsson A, Gabrielsson S, Nilsson B, Korsgren O (2003) Inflammatory mediators expressed in human islets of Langerhans: implications for islet transplantation. Biochem Biophys Res Commun 308: 474–479. 12914774 10.1016/s0006-291x(03)01392-5

[40] Mottram PL, Raisanen-Sokolowski A, Glysing-Jensen T, Stein-Oakley AN, Russell ME (1998) Redefining peripheral tolerance in the BALB/c to CBA mouse cardiac allograft model: Vascular and cytokine analysis after transient CD4 T cell depletion. Transplantation 66: 1510–1518. 9869093 10.1097/00007890-199812150-00015

[41] Zhu GQ, Wu ZF, Wang QT, Li YF (2004) Effect of IGF-1 gene transfected MSCs on the level of IGF-1, VEGF and BMP-2. Clin J Conserv 16: 371–374.

[42] Wang YL. Xu C, Sheng YH (2006) Effects of Epidermal Growth Factor on Cell Proliferation and Cell Cycle of Cultured Bovine Corneal Endothelial Cells in vitro. J Shanghai Jiaotong Univ 26: 877–879.

[43] Kutty RK, Nagineni CN, Kutty G, Hooks JJ, Chader GJ, et al (1994) Increased expression of heme oxygenase-1 in human retinal pigment epithelial cells by transforming growth factor-beta. J Cell Physiol 159: 371–378. 8163576 10.1002/jcp.1041590221

