# Correction: Combined Strategy of Endothelial Cells Coating, Sertoli Cells Coculture and Infusion Improves Vascularization and Rejection Protection of Islet Graft

**DOI:** 10.1371/annotation/0ab9adf8-8b5d-430b-87ec-6f760e72311d

**Published:** 2013-04-02

**Authors:** Yang Li, Wujun Xue, Hongbao Liu, Ping Fan, Xiaohong Wang, Xiaoming Ding, Xiaohui Tian, Xinshun Feng, Xiaoming Pan, Jin Zheng, Puxun Tian, Chenguang Ding, Xiaohu Fan

There was an error in the funding statement. The correct statement is:

This work was supported by grants from National Nature Science Foundation of China (Nos. 81270548, 81000309), the nation "973" project (2009CB522400), Science and technology project of Shaanxi (2012K16-09-02), Youth Foundation of the First Affiliated Hospital, Medical College, Xi'an Jiaotong University (2011YK.20). The funders had no role in study design, data collection and analysis, decision to publish, or preparation of the manuscript. 

