# Supplementary figures and images for: Combined Strategy of Endothelial Cells Coating, Sertoli Cells Coculture and Infusion Improves Vascularization and Rejection Protection of Islet Graft
Source: PLoS One. 2013 Feb 20;8(2):e56696. doi: 10.1371/journal.pone.0056696 (PMC3577699; doi:10.1371/journal.pone.0056696)

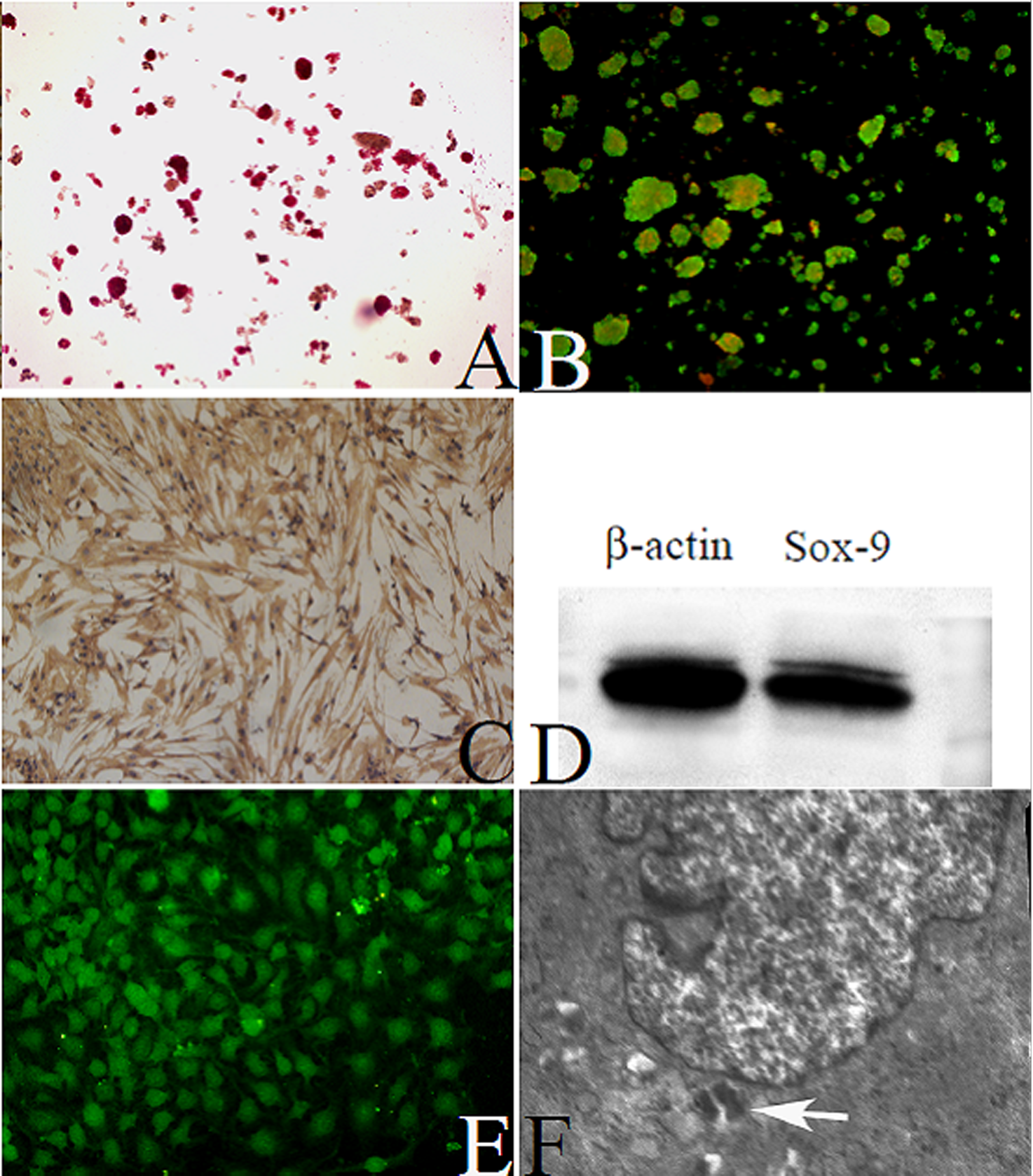

Supplement: Figure S1 — Islets, endothelial cells and Sertoli cells after separation and purification. (A) Optical microscopic observation of DTZ-stained islets. The islets appeared as scarlet, less acinar, and highly pure (magnification× 100). (B) Fluorescence microscopic observation of acridine orangepropidium iodide–stained islets. Most islets showed green fluorescence, indicating good activity (magnification×100). (C) Immunocytochemistry detected vimentin expression (magnification×100). (D) Western-blotting detected Sox-9 expression in cultured Sertoli cells after isolation. (E) Immunofluorescence detected vWF expression (magnification×100). (F) ECs were observed by TEM. White arrows point to Weibel-Palade bodies which was a characteristic of ECs. (magnification×10000). (TIF) [file pone.0056696.s001.tif]

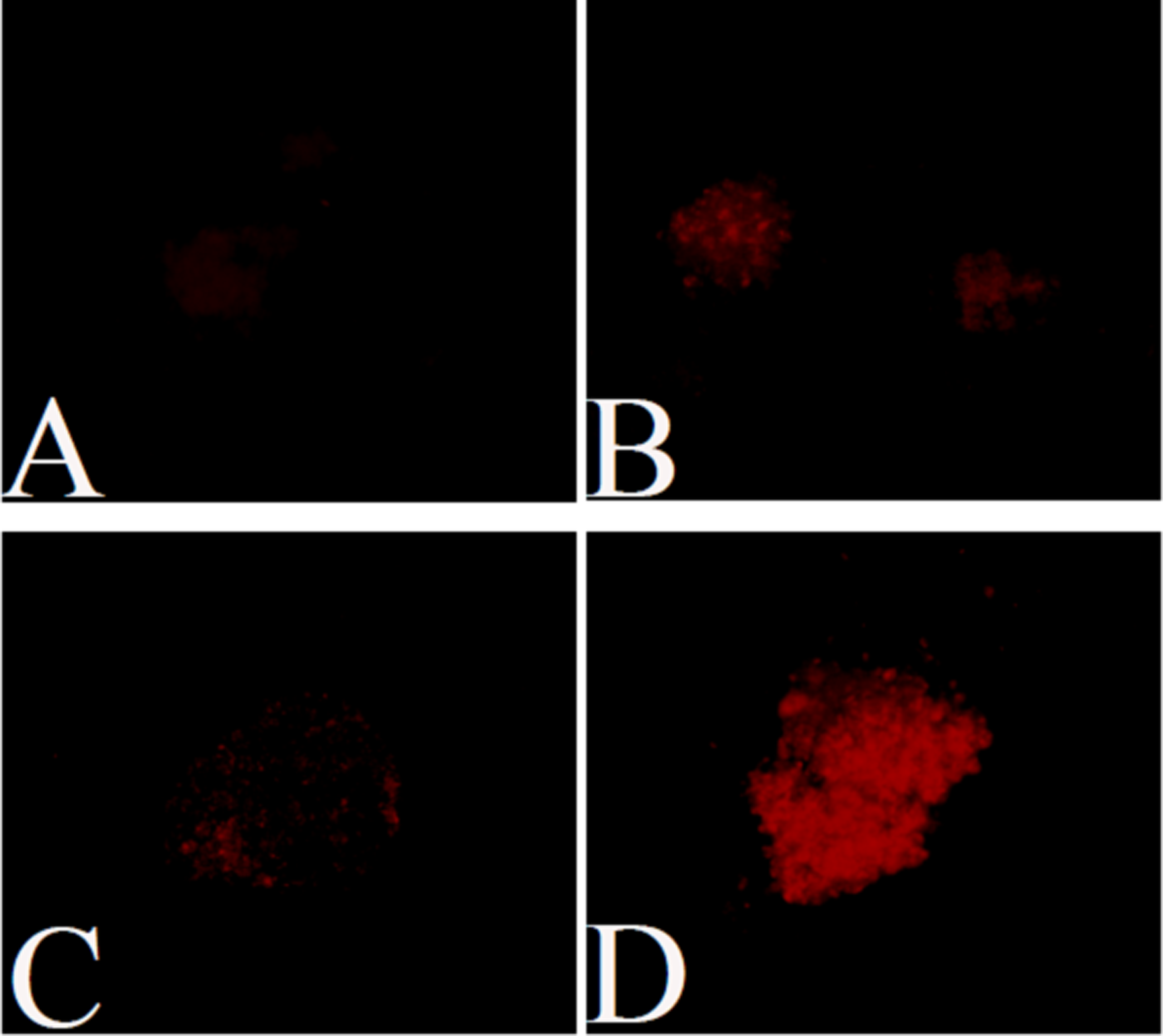

Supplement: Figure S2 — Detection of vWF in islets after culture by immunofluorescence 7 days after culture. (A) In control group, the expression of vWF was nearly undetectable 7 days after culture; (B) In the ECs-coated group, small amount of ECs around islets, which is significantly less than those of groups D; (C)In Sertoli cells co-cultured group, there were nearly no ECs around islets. (D) In ECs-coated islets and Sertoli cells coculture group, large number of vWF-positive ECs around islet. Magnification×200. (TIF) [file pone.0056696.s002.tif]

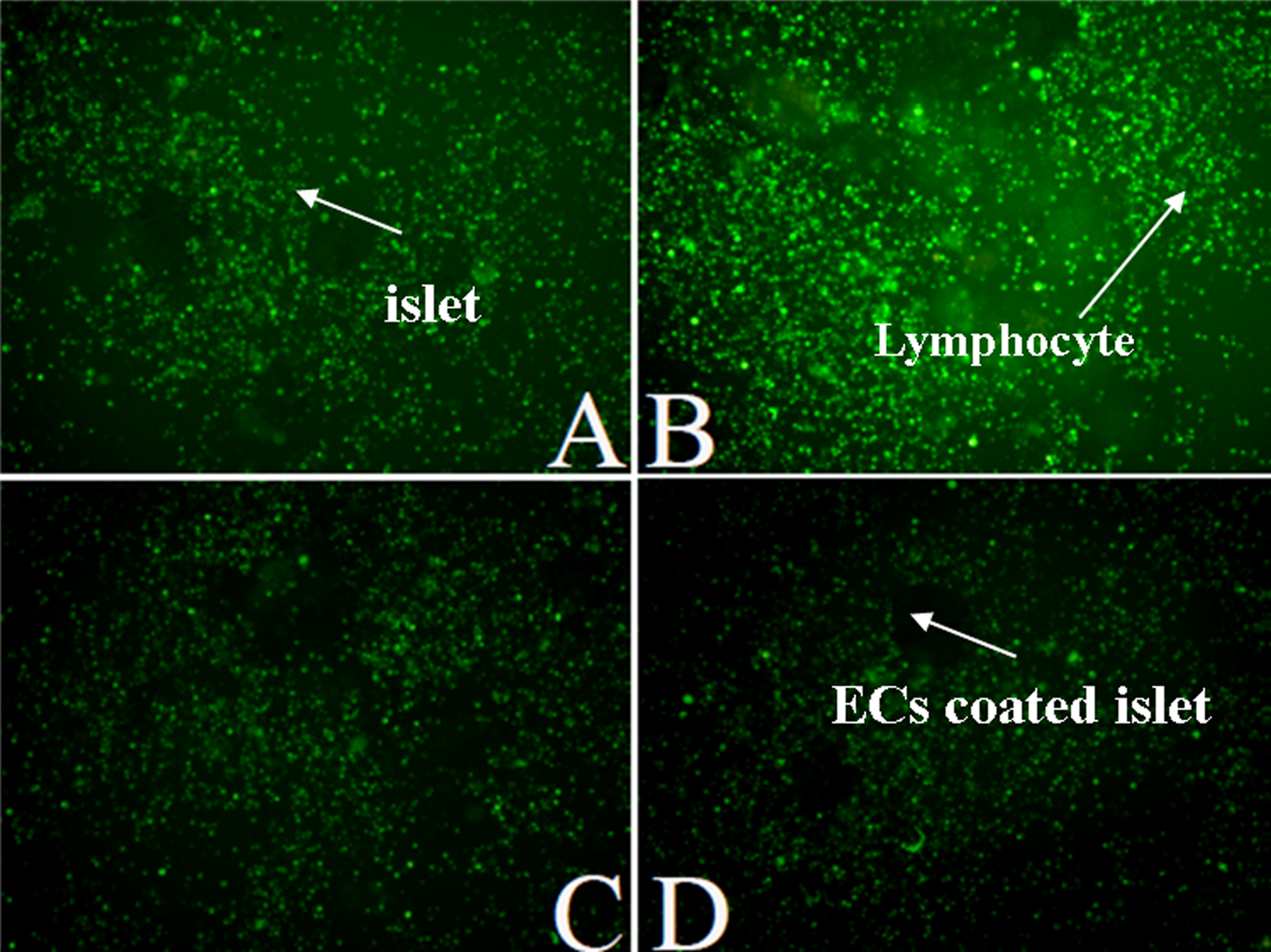

Supplement: Figure S3 — MLR and proliferation index in each group. Lymphocyte mixed with (A)islets; (B)ECs-coated islets; (C)Islets co-cultured with Sertoli cells. (D)ECs-coated islets co-cultured with Sertoli cells. Magnification×100. (TIF) [file pone.0056696.s003.tif]
