# Supplementary material for: Combined Strategy of Endothelial Cells Coating, Sertoli Cells Coculture and Infusion Improves Vascularization and Rejection Protection of Islet Graft
Source: PLoS One. 2013 Feb 20;8(2):e56696. doi: 10.1371/journal.pone.0056696 (PMC3577699; doi:10.1371/journal.pone.0056696)
Supplement: Table S1 — Insulin release and stimulation index (SI) by glucose-stimulation test in four groups at different culture time. (DOC) [file pone.0056696.s004.doc]

**Table S1. Insulin release and stimulation index (SI) by glucose-stimulation test in four groups at different culture time.**
