# Supplementary material for: Combined Strategy of Endothelial Cells Coating, Sertoli Cells Coculture and Infusion Improves Vascularization and Rejection Protection of Islet Graft
Source: PLoS One. 2013 Feb 20;8(2):e56696. doi: 10.1371/journal.pone.0056696 (PMC3577699; doi:10.1371/journal.pone.0056696)
Supplement: Table S2 — Cytokines secretion profiles in culture media in each group. (DOC) [file pone.0056696.s005.doc]

**Table S2. Cytokines secretion profiles in culture media in each group**

*** P<0.05 vs. group A, ^ P<0.05 vs. group B, # P<0.05 vs. group C.**
